# In vivo label-free mapping of the effect of a photosystem II inhibiting herbicide in plants using chlorophyll fluorescence lifetime

**DOI:** 10.1186/s13007-017-0201-7

**Published:** 2017-06-15

**Authors:** Elizabeth Noble, Sunil Kumar, Frederik G. Görlitz, Chris Stain, Chris Dunsby, Paul M. W. French

**Affiliations:** 1 Photonics Group, Department of Physics, Imperial College London, London, SW7 2AZ UK; 2 Department of Chemistry, Imperial College London, London, SW7 2AZ UK; 3 Institute of Chemical Biology, Imperial College London, London, SW7 2AZ UK; 4 Syngenta, Jealott’s Hill International Research Centre, Bracknell, Berkshire RG42 6EY UK; 5 Centre for Pathology, Imperial College London, London, SW7 2AZ UK

**Keywords:** Fluorescence spectroscopy, Plant, FLIM, Herbicide, Photosystem II, Chlorophyll fluorescence lifetime

## Abstract

**Background:**

In order to better understand and improve the mode of action of agrochemicals, it is useful to be able to visualize their uptake and distribution in vivo, non-invasively and, ideally, in the field. Here we explore the potential of plant autofluorescence (specifically chlorophyll fluorescence) to provide a readout of herbicide action across the scales utilising multiphoton-excited fluorescence lifetime imaging, wide-field single-photon excited fluorescence lifetime imaging and single point fluorescence lifetime measurements via a fibre-optic probe.

**Results:**

Our studies indicate that changes in chlorophyll fluorescence lifetime can be utilised as an indirect readout of a photosystem II inhibiting herbicide activity in living plant leaves at three different scales: cellular (~μm), single point (~1 mm^2^) and macroscopic (~8 × 6 mm^2^ of a leaf). Multiphoton excited fluorescence lifetime imaging of *Triticum aestivum* leaves indicated that there is an increase in the spatially averaged chlorophyll fluorescence lifetime of leaves treated with Flagon EC—a photosystem II inhibiting herbicide. The untreated leaf exhibited an average lifetime of 560 ± 30 ps while the leaf imaged 2 h post treatment exhibited an increased lifetime of 2000 ± 440 ps in different fields of view. The results from in vivo wide-field single-photon excited fluorescence lifetime imaging excited at 440 nm indicated an increase in chlorophyll fluorescence lifetime from 521 ps in an untreated leaf to 1000 ps, just 3 min after treating the same leaf with Flagon EC, and to 2150 ps after 27 min. In vivo single point fluorescence lifetime measurements demonstrated a similar increase in chlorophyll fluorescence lifetime. Untreated leaf presented a fluorescence lifetime of 435 ps in the 440 nm excited chlorophyll channel, CH4 (620–710 nm). In the first 5 min after treatment, mean fluorescence lifetime is observed to have increased to 1 ns and then to 1.3 ns after 60 min. For all these in vivo plant autofluorescence lifetime measurements, the plants were not dark-adapted.

**Conclusions:**

We demonstrate that the local impact of a photosystem II herbicide on living plant leaves can be conveniently mapped in space and time via changes in autofluorescence lifetime, which we attribute to changes in chlorophyll fluorescence. Using portable fibre-optic probe instrumentation originally designed for label-free biomedical applications, this capability could be deployed outside the laboratory for monitoring the distribution of herbicides in growing plants.

**Electronic supplementary material:**

The online version of this article (doi:10.1186/s13007-017-0201-7) contains supplementary material, which is available to authorized users.

## Background

Understanding the specific fates of agrochemicals in plants is crucial to optimise their function. The absorption, distribution, transportation and storage of agrochemicals within and through the various cellular structures will greatly affect the extent and effectiveness of their interaction with plant metabolism [1] and so the ability to map the distribution or effect of agrochemicals in vivo could help optimise the formulation of new agrochemicals.

In vitro methods that are based on solvent extraction or cell culture analysis, such as biochemical assays or mass spectrometry [2, 3], can provide highly specific chemical information but are destructive and time consuming. Furthermore, any physical or chemical perturbation to the natural state of a plant can result in stress, potentially triggering various reflex mechanisms that could lead to incorrect inferences [4] and such measurements can fail to report the true spatio-temporal distribution and interaction of agrochemicals. Analytical methods such as radiolabelling [5] can provide highly specific information on herbicide metabolism and over all absorption rates, but do not offer spatially resolved herbicide distribution data. Scanning electron microscopy (SEM) [6] can provide higher spatial resolution but can be prone to artefacts arising from sample fixation and mechanical sectioning and cannot follow dynamic events.

Fluorescence imaging techniques, such as wide-field fluorescence imaging [7], confocal laser scanning microscopy (CLSM) [8, 9] and two-photon excitation microscopy (TPEM) [10, 11], provide the ability to follow dynamic events, including in live plants. Hence, it is desirable to explore non-invasive fluorescence imaging techniques, capable of providing qualitative and quantitative information concerning the in vivo distribution of agrochemicals, in the laboratory and in the field. In principle, this could be realised by fluorescence imaging of agrochemicals that are intrinsically fluorescent. However, it is generally challenging to distinguish such fluorescence from background autofluorescence arising from chlorophyll and/or other endogenous fluorophores. Direct fluorescent labelling of the agrochemical could improve the contrast but such labels are comparable to or larger in size than the agrochemicals themselves and may compromise their mode of action. An alternative approach is to map the local action of an agrochemical through its impact on the intrinsic (chlorophyll) fluorescence and thereby infer information concerning the agrochemical distribution. Here we demonstrate this approach using fluorescence lifetime imaging of chlorophyll to map the local action of a PS II inhibiting herbicide.

Herbicides represent a major fraction of all agrochemicals used, of which PS II inhibiting herbicides are an important class. Light energy absorbed by leaves can be expended driving the photochemistry of photosynthesis, emitted as chlorophyll fluorescence or dissipated as heat [12]. When a chlorophyll molecule absorbs light and is promoted to its first singlet excited state, the excited state energy can be transferred to other chlorophyll molecules and ultimately to the photosynthetic reaction centres (PS II and PS I) through Förster resonance energy transfer [13]. The presence of a photosynthetic inhibitor suppressing this pathway leads to an increase in the lifetime of singlet excited chlorophyll, which increases the probability of inter-system crossing to long lived chlorophyll triplet excited states. This increases the probability of transfer of energy from the triplet excited excited states of chlorophyll to ground state molecular oxygen, which results in the formation of singlet state molecular oxygen that can subsequently lead to the production of radical species such as ^3^Chl, ^1^O_2_, H_2_O_2_ and O_2_^−^. These radicals are phytotoxic in nature and can hinder important biological processes or cause membrane damage. Treating a plant with a PS II inhibitor will trigger an increased production of such radical species and can result in phytotoxicity. This is the basis for the mode of action of most of PS II inhibiting herbicides [14].

Chlorophyll fluorescence can thus provide a readout of the action of PS II inhibitors. In general, the balance between photochemistry, chlorophyll fluorescence and heat dissipation following the absorption of light has been studied using a range of chlorophyll fluorometric techniques that are sensitive to the photosynthetic electron transfer rate and therefore provide information on the overall PS II efficiency [15]. Early studies were based on the assumption that the light energy absorbed in leaves was divided equally between the PS I and PS II light harvesting complexes [15] and therefore the factor, *F*_*v*_/*F*_*m*_, which is the ratio of fluorescence variation to maximal fluorescence, gives a measure of maximum PS II efficiency [16]. Note that *F*_*v*_ = *F*_*m*_ − *F*_*o*_, where *F*_*o*_ is the fluorescence from the leaf in the presence of weak measuring light (~0.1 µmol photons m^−2^ s^−1^) and *F*_*m*_ is the maximum fluorescence from a dark-adapted leaf when excited by a saturating light flash, i.e. one with sufficient intensity to drive a high proportion of the PSII centres into the “closed” state where they are not capable of photochemistry. For Arabidopsis, a saturating photon flux density of ~4000 μmol photons m^−2^ s^−1^ is required [17] but this value may vary between plant species. Subsequent studies, however, showed that this assumption was not valid due to PS I fluorescence varying independently of photosynthetically active photon flux density, unlike PS II [15]. Furthermore, the PSI and PSII fluorescence vary differently as a function of temperature [18]. Therefore estimates of the photosynthetic electron transfer rate based on measurements of photosynthetic yield, *F*_*v*_ */F*_*m*_ [17] could be erroneous. Nevertheless, chlorophyll fluorescence-based approaches have been successfully applied at different scales to study the dynamics of basic photosynthetic reactions, including biotic/abiotic stress responses [19–21], and efforts are being made to translate this optical signal from laboratory to field phenotyping [22] in plants, linking microscopic observations to macroscopic and to leaf level dynamics of photosynthetic reactions. Chlorophyll fluorescence imaging has previously been used to obtain qualitative readouts of metabolic changes correlated to herbicide action, as reported in [17], although the instrumentation used could not localise herbicide distribution with high resolution. Higher resolution fluorescence imaging studies [15, 23, 24], have demonstrated that the uptake of the herbicide Diuron can be monitored in plant leaves using chlorophyll fluorescence imaging following dark adaption, although these utilised wide-field imaging studies and so did not provide depth-resolved imaging of changes to chlorophyll fluorescence. Optically sectioned imaging can be provided by confocal laser scanning fluorescence microscopy and used to image up to a depth of 100–150 µm but image quality deteriorates when imaging deeper than ~100 µm into the sample owing to background fluorescence and scattering effects caused by the leaf tissues [25]. Improved performance in terms of imaging depth and reduced background fluorescence can be provided by two photon excitation, due to the increased ability of near-infrared light to penetrate biological samples and the limiting of excitation to the focal plane, as has been applied in plant leaves e.g. [26]. However, optical scattering and aberrations still impact the quantification of intensity-based chlorophyll fluorescence readouts.

Fluorescence lifetime measurements [27] can provide quantitative readouts even when image information is degraded and absolute intensity measurements are compromised by optical scattering, sample absorption (inner filter effect) and/or variations in fluorophore concentration. Fluorescence lifetime provides a direct readout of the impact of the local fluorophore environment on relaxation pathways following excitation and so provides powerful sensing capabilities. In biomedicine, fluorescence lifetime measurements and fluorescence lifetime imaging (FLIM) are used to study changes in tissue autofluorescence [28]. However, fluorescence lifetime techniques have not been widely utilized for plant studies although FLIM has been used to study photosynthesis [26, 29, 30], and the uptake of minerals [31, 32] and was previously used to study the effect of a photosynthetic inhibitor DCMU (3-(3,4-dichlorophenyl)-1,1-dimethylurea) in *Chlamydomonas reinhardtii* [29]. We report here the application of fluorescence lifetime imaging of chlorophyll to provide a label-free in vivo means to non-invasively map the effect of a PS II inhibiting herbicide in *Triticum aestivum* (Winter Wheat) on different spatial scales through its local impact on chlorophyll fluorescence.

The action of inhibitors on the photosynthetic electron transport chain has previously been observed using non-imaging, time-resolved fluorescence spectroscopy techniques: Petrasek et al. [33] observed an overall increase in chlorophyll fluorescence lifetime under the stress of a PS II inhibitor, DCMU, and Hunsche et al. [34] reported a significant increase in the mean lifetime of the fluorescence measured from plants treated with PS II inhibiting herbicides. FLIM experiments have also shown that the inhibition of PS II by a herbicide leads to an increase of the chlorophyll fluorescence intensity and lifetime—attributed to a reduction in photochemical quenching—but no spatially resolved lifetime data was presented [26].

Here we explore the potential to utilise the change in chlorophyll fluorescence lifetime as an indirect read out of herbicide activity and thereby map the time-dependent uptake of the herbicide by plants in vivo—and ultimately in the field—using FLIM or single-point probe lifetime measurements. We present in vivo readouts from non-dark adapted plants at three different scales: cellular (~μm), single point (~1 mm^2^) and macroscopic (~8 × 6 mm^2^ of a leaf) to study the distribution of the effect of a PS II inhibiting herbicide. We first utilised two-photon laser scanning microscopy (TPLSM) to perform in vivo FLIM in leaves excited at 900 nm, taking advantage of the deeper optical sectioning and reduced out-of-plane photobleaching and phototoxic effects compared to single photon laser scanning confocal microscopy [35]. While it is difficult to make a generalization of the typical imaging depth achievable, since plant leaves have a complex structural anatomy that varies from species to species, we note that TPLSM imaging depths of ~200 μm have been reported [10, 36]. We also explored the potential to repurpose instrumentation originally developed for autofluorescence lifetime studies of human tissue for medical applications, noting that this instrumentation is already relative portable and could be engineered for application in the field. Specifically, we applied a custom-built time-resolved spectrofluorometer, which was originally developed to make single point measurements of tissue via a fibre-optic probe [37] to make measurements of plant leaves treated and untreated with the PSII inhibiting herbicide, Flagon 400 EC. This approach can provide mapping of the action of herbicides with a spatial resolution on the order of millimetres. Finally, for macroscopic imaging, we applied wide-field time-gated FLIM technology [38], obtaining maps of herbicide action on a time scale of seconds. We note that spectrally resolved lifetime measurements can provide further contrast, which could address the interference from other plant pigments, e.g. [39, 40].

## Methods

### Plant material and growth conditions

For the studies conducted, plants of *Triticum aestivum* (Winter Wheat cv. Hereward), a cool climate monocot food crop (Growth room conditions: 20/16 °C (day/night temp) 16 h of daylight, approx. 65% relative humidity, lighting-150 μmol/m^2^/s or 31.2 W/m^2^) were used. Seeds were obtained from Syngenta Jealott’s Hill International Research Centre Bracknell, Berkshire, UK.

### Chemicals

PS II inhibiting herbicide Flagon 400 EC was provided in solution by Syngenta (Jealott’s Hill International Research Centre Bracknell, Berkshire, UK) and was diluted in milliQ water.

### Sample preparation and plant treatment

Before the plants are treated with active ingredient (AI) formulation, a non-fluorescent felt pen (Berol Toughpoint) was used to mark the desired treatment area with four spots on a square with side approximately 10 mm so that the treated area could be easily located again in subsequent fluorescence measurements. Flagon EC 400 was diluted in water to a concentration of ~49 ppm/1.22 M and two closely spaced 2 μl droplets (a close approximation to the the size of typical spray droplets used in the field) were applied using a Hamilton micropipette (Hamilton Bonaduz AG, Bonaduz, Switzerland) in the centre of the four marked spots.

For measuring the leaf autofluorescence excitation/emission matrix, a new leaf was used for each emission spectrum to avoid any photochemical changes to the leaf caused by the excitation light. For each set of experiments, all the leaves were from plants of the same age and grown under the same growth conditions. Unless otherwise specified, we used 18 days old plants for the measurements reported here.

### Measurement of fluorescence spectra

The autofluorescence of *Triticum aestivum* plant leaves and also the fluorescence properties of the herbicide were characterised in order to optimise the excitation wavelengths and fluorescence detection spectral windows for these studies. The fluorescence excitation and emission spectra of Flagon EC 400 and the plant leaves were measured using a commercial UV/VIS Spectrofluorometer (RF-5301PC, Shimadzu, Japan).

### Characterisation of Flagon 400 EC fluorescence

Time-resolved fluorescence measurements of Flagon EC 400 were undertaken using the custom-built multidimensional spectrofluorometer described in [41], of which a schematic diagram of the optical set-up of the system is provided in Additional file 1. A supercontinuum laser source (SPC-400, 20 MHz Fianium, UK) operating at a repetition rate of 20 MHz provides tunable picosecond pulses for excitation. For measurements of Flagon EC 400, a bandpass filter centered at 400 and 40 nm bandwidth was used to select the excitation radiation that passes through a polarizer before reaching sample solution in a cuvette. The detection beam path is at right angles to the excitation beam path and the resultant fluorescence decay is recorded for 11 distinct emission wavelengths spaced 2 nm apart over the range 480–500 nm, using time-correlated single photon counting (TCSPC), with an average of ~6000 photon counts over an acquisition time of 180 s per decay. All measurements were performed with a polarizer in the emission path placed at the magic angle polarization to remove any fluorescence anisotropy effects. The whole system is controlled by a custom-written LabVIEW software (LabVIEW, National Instruments). The instrument response function (IRF) for the fluorometer is measured using a scatterer, LUDOX (a solution of colloidal silica, Sigma-Aldrich, UK). The fluorescence decay curves are analysed using the *FLIMfit* software tool developed at Imperial College London [42] and the maximum likelihood iterative method was used to fit the experimental data to model fluorescence decay profiles.

### Multiphoton excited fluorescent lifetime imaging (MPE-FLIM)

MPE-FLIM in plant cells was undertaken both in vivo in a live plant leaf and in situ in a recently removed plant leaf in order to obtain high (subcellular) resolution maps of chlorophyll fluorescence lifetime. FLIM measurements were made using a Leica SP5 system (TCS SP5, Leica Microsystems GmbH, Germany) with a tunable (690–1020 nm) Ti:Sapphire laser (Spectra-Physics, Broadband Mai Tai) providing 100 fs pulses at 80 MHz for multiphoton excitation and FLIM implemented with TCSPC. The optimum excitation wavelength for mulitphoton imaging of chlorophyll in a plant leaf was determined using a detection band of 680–735 nm and by scanning the excitation wavelength over the range 850–990 nm. The highest signal was obtained for excitation at 900 nm and so this was chosen for all subsequent multiphoton imaging. Treated and untreated leaf samples were imaged in situ and in vivo using multiphoton excitation at 900 nm and detection in the band 600–730 nm, with typical acquisition times of 40 s being required to acquire images with 256 × 256 pixels. The leaves were treated with two closely spaced 2 μl droplets of Flagon EC 400 and were imaged after 2.5 h for the in situ experiment. For in vivo time course experiments, images were taken at 5 min intervals starting from 5 min after treatment to 45 min.

The instrument response function (IRF) of the system was recorded using gold nano-rods and a background image (with no excitation) with the same experimental parameters as FLIM measurements. The fluorescence decay data were analyzed using the *FLIMfit* software [42].

### Multispectral point-probe fluorescence lifetime measurements in vivo

**Fig. 1:**
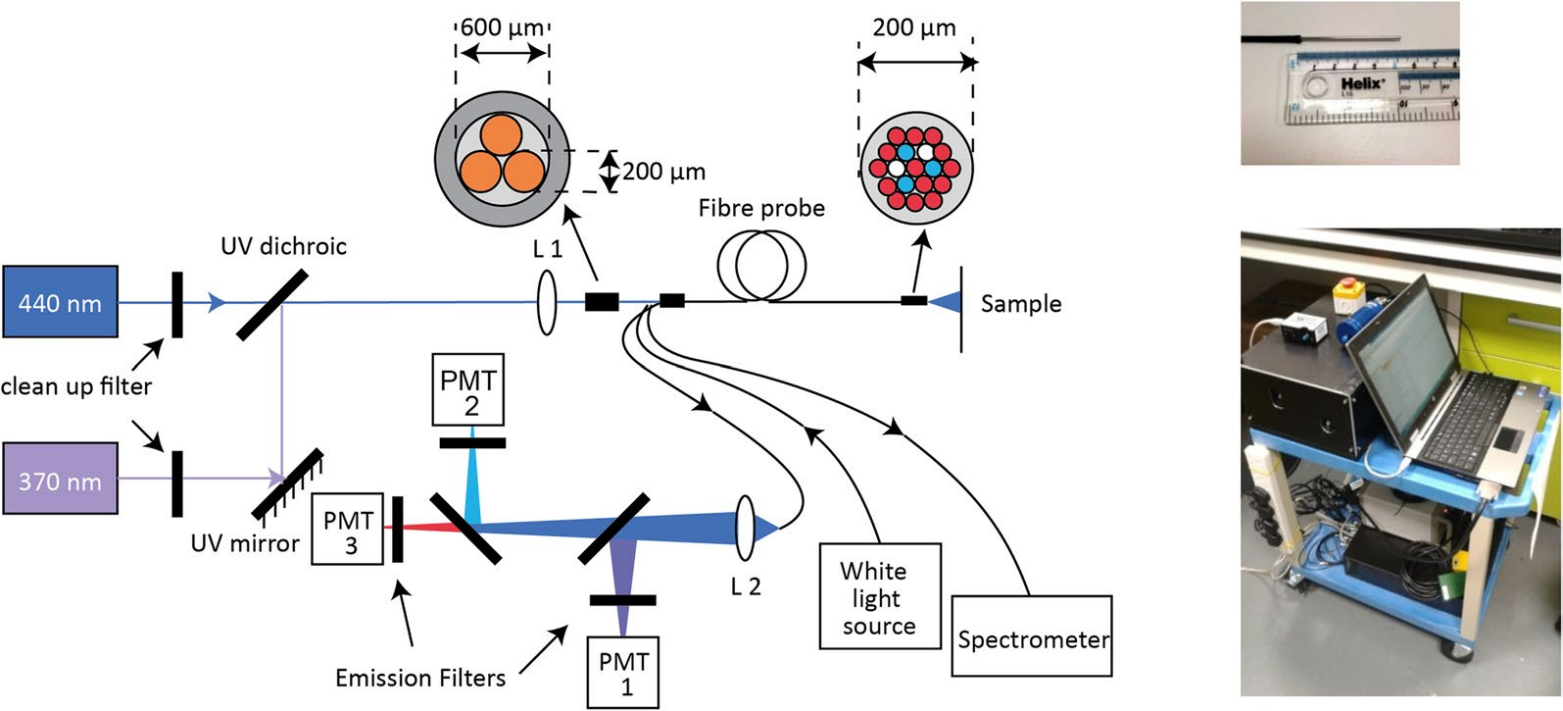
Schematic diagram of portable multispectral time-resolved spectrofluorometer with fibre-optic probe with inset of probe tip and photograph of instrument

To explore the potential to monitor herbicide distribution in the field, we used a portable time-resolved spectrofluorometer incorporating a fibre-optic probe that was originally developed for clinical and preclinical studies [37], to make single point measurements of leaves on live plants. This instrument, which is depicted in Fig. 1, incorporates two picosecond pulsed excitation diode lasers: a laser diode (LDH-P–C-375B, PicoQuant GmbH, Germany) that provides 70 ps pulses at 372 nm with an average output power of 3.3 mW and a laser diode (LDH-P–C-440B, PicoQuant GmbH, Germany) providing 90 ps pulses at 440 nm with 3.5 mW average power. The repetition rates of both the lasers were set to 20 MHz. The laser beams are coupled into a custom-made optical fiber bundle (FiberTech Optica, Canada) consisting of three excitation fibers and fourteen detection fibers arranged in a hexagonal structure around the excitation fibers.

For these experiments the fibre-optic probe was held by a clamp to the leaf and fluorescence was collected by the detection fibers into three spectrally resolved detection channels implemented using a set of dichroic mirrors and band-pass filters. The leaves were treated with two drops (2 μl) of the herbicide Flagon EC 400 and measurements were made 5 min afterwards. At 372 nm excitation, all the three channels are used: the first channel collects light from 400 to 420 nm (CH1); the second channel collects fluorescence light from 430 to 480 nm (CH2); and the third channel collects light from 620 to 710 nm (CH3). For the 440 nm excitation light, only the third (620–710 nm) channel is active and this is referred as “channel 4” (CH4). Since 440 nm is an optimum excitation wavelength for chlorophyll fluorescence in leaves, CH4 is the spectral channel of primary interest for this study. The integration time for each acquisition was about 30 s for both UV excitation as well as 440 nm excitation. 1.7 μW of 375 nm excitation and 29 μW of 440 nm excitation at the distal tip of the fibre probe were used for measurements of plant leaves. IRF measurements were made for each detection channels using a scattering sample under the same conditions. This fibre-optic probe interrogates an area of ~1 mm^2^ on the leaf and so can provide mm spatial resolution.

### In vivo wide-field macroscopic time-gated fluorescence imaging

**Fig. 2:**
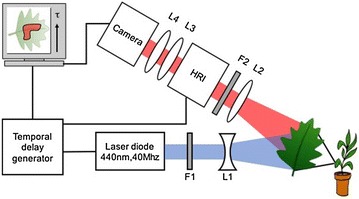
Schematic diagram of the wide field imaging macroscope. 440 nm pulsed laser beam is passed through a cleanup filter FI and expanded using a diverging lens, L1 to illuminate a leaf. Fluorescence from the leaf is collected by a camera lens, L2, and passed through an emission filter, F2, before it reaches a high rate imager (HRI). The collected signal is imaged on to a sCMOS camera by a pair of relay lenses L3, L4. The HRI is synchronized with the excitation laser via a temporal delay generator

For imaging herbicide distribution with higher spatial resolution and on faster timescales, we constructed a wide field FLIM macroscope for in vivo imaging of plants. A schematic diagram describing the setup is shown in Fig. 2. Our system design partially follows previously described wide field FLIM endoscope instrumentation [43, 44], but here free space illumination using a diverging lens was used for conveniently exciting the leaf samples. The excitation source was a gain-switched diode laser (PicoQuant, LDH-P–C-440B with driver PDL-800-B), which provided pulses of <500 ps at 40 MHz with average powers of ~4 mW. The emission from the diode laser was passed through a spectral clean-up filter (F1, Semrock, FF02-438/24) to suppress out of band radiation from the diode laser and the elliptical beam was expanded by a diverging lens to illuminate a FOV of ~30 × 6 mm on a leaf held in place by a clamp while attached to a live plant. A black anodized piece of metal was fixed to the clamp behind the leaf to block any unwanted signals or reflections. Neutral density filters were inserted inbetween the clean-up filter and diverging lens to reduce the excitation beam power to 0.3 mW. The fluorescence from the leaf was collected by a camera lens (Pentax 12.5–75 mm) and passed through an emission filter (F2, Semrock, 641/75 nm bandpass filter) chosen to overlap the chlorophyll emission peak at 690 nm. The filtered fluorescence signal was focused onto a gated optical intensifier (Kentech Instruments, model HRI) read out by a sCMOS camera (Andor, Zyla 5.5) via two relay lenses (L3, L4). Wide-field time-gated FLIM, entails synchronizing the pulsed laser source with the HRI and adjusting the delay between excitation and time-gated detection to sample the fluorescence intensity decay profiles in each pixel. For this study we sampled the fluorescence signal with 9 time gates of 1 ns duration at increasing time delays after the excitation pulse. The read out camera was operated with an exposure time of 200 ms per frame. Overall, each FLIM acquisition required ~3 s.

The leaf sample was first imaged once without any treatment and then a time-series of FLIM images was acquired at 3 min intervals after application of two 2 μl drops of Flagon EC 400. Image acquisition was controlled by the openHCA-FLIM μManager plug-in developed in the Photonics group at Imperial College London [45]. An IRF based on the excitation pulses was measured under the same experimental conditions using a scattering sample.

## Results

### Plant autofluorescence characterization

**Fig. 3:**
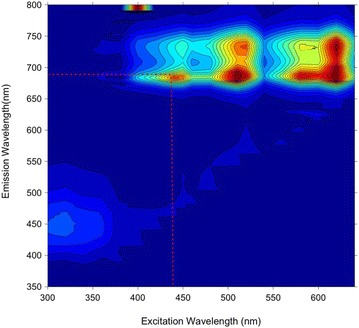
Excitation emission matrix of fluorescence from *Triticum aestivum* leaves measured using a spectrofluorometer. Fluorescence excitation was scanned over a wavelength range 300–650 nm at 10 nm intervals and corresponding emission spectrum measured for each excitation wavelength. A different leaf was used for each emission scan

The fluorescence excitation-emission matrix for *Triticum aestivum* leaves measured using the Shimadzu spectrofluorometer is shown in Fig. 3. There are two major spectral emission bands: a blue-green fluorescence (ex ~320 nm, em ~450 nm) that could be due to the presence of cinnamic acids [46] and a fluorescence band attributed to chlorophyll with two prominent peaks at 690 nm and 720 nm [47]. For the subsequent fibre optic probe and wide-field imaging fluorescence lifetime measurements discussed below, we chose to concentrate on the 690 nm emission peak attributed to fluorescence from chlorophyll in leaves at 440 nm excitation as the spectral region of interest. This matched the capabilities of our instrumentation and we detected no significant fluorescence from the Flagon EC 400 in this spectral detection band, see below.

### Characterisation of Flagon EC 400 fluorescence

**Fig. 4:**
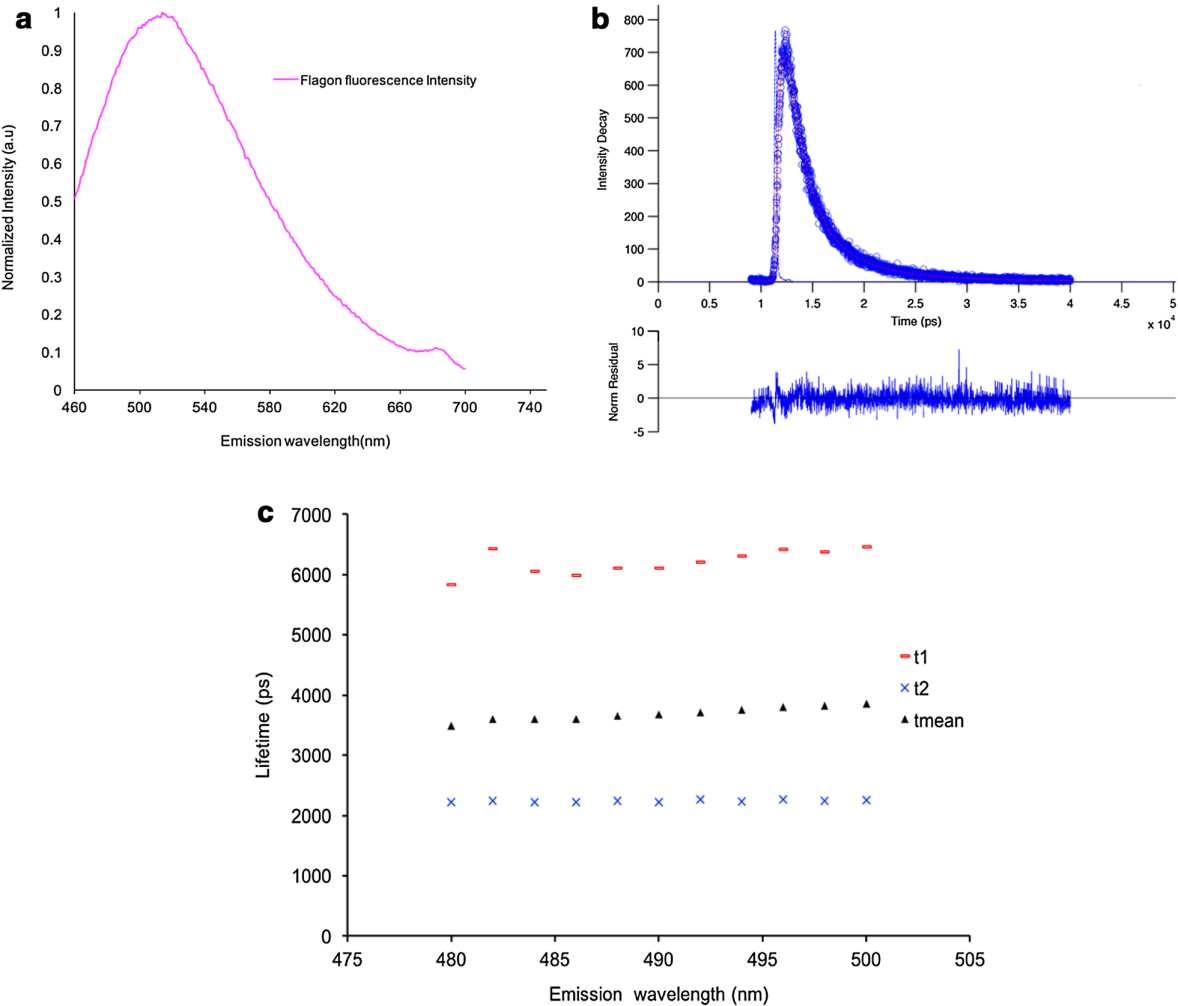
**a** Fluorescence emission spectrum of Flagon EC 400 when excited at 440 nm. **b** Fluorescence decay curve fitted with double exponential model using the FLIMfit software; **c** dependence of fluorescence lifetime on detection wavelength. Data shown in **b** and **c** was acquired with an excitation wavelength of 405 nm

The emission spectrum of Flagon EC 400 in the range 400–800 nm was measured (49 ppm solution) using the Shimadzu spectrofluorometer with excitation at 440 nm, see Fig. 4a. The fluorescent decay profiles of Flagon EC 400 were measured (49 ppm solution) using the custom-built multidimensional spectrofluorometer as described in Manning et al. [41] with excitation at 405 nm (20 MHz repetition rate) and detection at 482 nm. The data were fitted to a double exponential decay model using *FLIMfit* which returned lifetime components of 6214 ps (18%) and 2239 ps (82%) with an average χ^2^ value of 1.17. An exemplar decay profile is shown in Fig. 4b. We investigated how the fluorescence decay profile varied across the emission spectrum and the results are presented in Fig. 4c. Both lifetimes remain approximately constant over the detection range 480–500 nm.

**Fig. 5:**
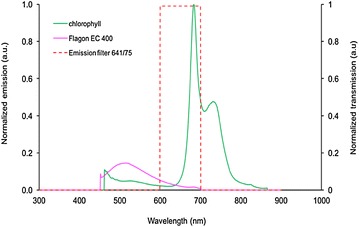
Fluorescence emission spectra of chlorophyll and Flagon EC 400, when excited at 440 nm (curve normalized to maximum Flagon fluorescence). The transmission band of the emission filter used for wide-field FLIM is also plotted to show the spectral range of detection. The emission curves are scaled according to the data from control experiments carried out using the multispectral point-probe spectrofluorometer

In order to show that fluorescence from Flagon EC400 is small compared to that from the *Triticum aestivum* leaf when using fluorescence excitation at 440 nm, the two emission spectra were scaled according to the measurements of their relative emission intensities using the multispectral point-probe spectrofluorometer, see Fig. 5. Here the emission intensity of Flagon EC 400 was measured when it was dried on to a black anodised aluminium surface. From this data the relative fluorescence signal from Flagon EC 400 is only 0.58% of chlorophyll fluorescence from a leaf obtained in the same spectral channel.

### In vivo multiphoton FLIM showing effect of herbicide at a cellular level

**Fig. 6:**
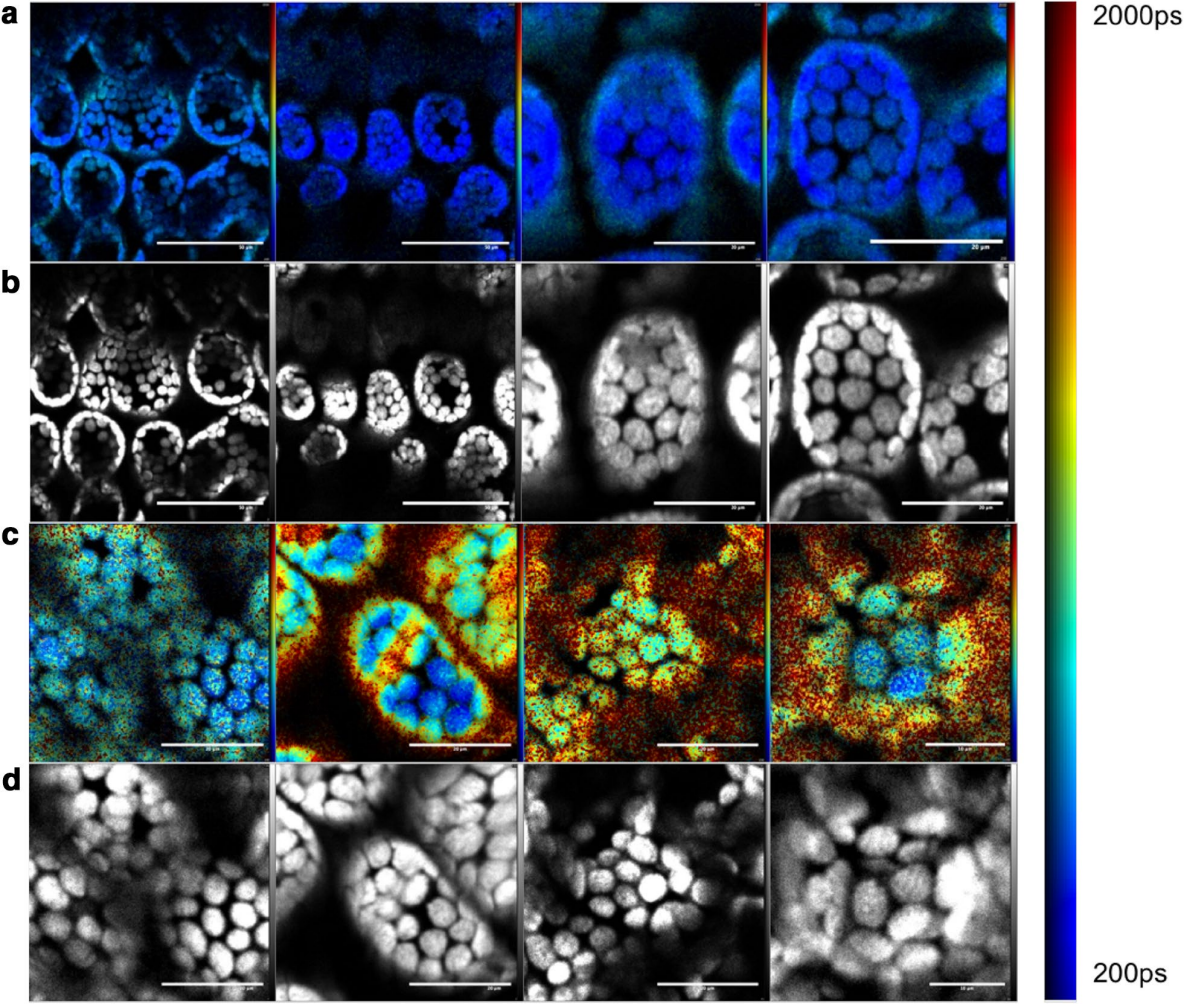
TPE-FLIM images of *Triticum aestivum* obtained using a Leica TCS SP5 in situ. *Row* **a** shows fluorescence intensity images of the mesophyll cell layer in an untreated wheat leaf (different columns show different FOVs in the same leaf). *Row* **b** shows the corresponding false-colour intensity-weighted mean lifetime (*τ*_m_). *Row* **c** shows fluorescence intensity images from different FOVs of a wheat leaf treated with two 2 μl drops of Flagon EC 400 and imaged after 2.5 h. *Row* **d** shows the corresponding *τ*_m_ images. The two-photon excitation wavelength was 900 nm, emission was collected in the range 600–730 nm and a ×40 air objective with an NA of 0.75 was used for imaging. *Scale bar* 50 μm for *columns* 1, 2 and 20 μm for *columns* 3,4

**Fig. 7:**
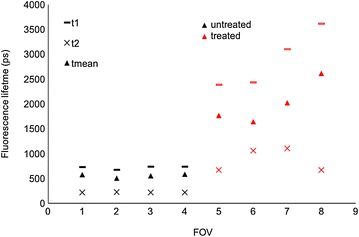
Fluorescence lifetimes (*τ*_m_) calculated from different FOVs in treated and untreated leaves. (1–4) Represent FOVs in the untreated leaf and (5–9) represents FOVs in a treated leaf. The data is obtained from the TPE-FLIM measurements shown in Fig. 6

To explore the indirect read out of the presence of the Flagon EC 400 herbicide, we used two photon excitation at 900 nm to acquire high resolution FLIM images of chlorophyll fluorescence in *Triticum aestivum* leaves. Imaging of treated (Flagon EC 400) and untreated plant leaves was performed in situ using TCSPC on the Leica SP5 multiphoton system with a detection spectral band from 600 to 730 nm and implemented with leaf samples from the same plant fixed to a microscope slide. We imaged 4 fields of view (FOVs) in the sample of untreated leaf and 4 FOVs in the sample of treated leaf at 2.5 h post treatment. Figure 6a, c show typical autofluorescence intensity images and Fig. 6b, d show the corresponding intensity-merged fluorescence lifetime images of the untreated and treated leaves respectively. These images show mesophyll cells (larger ovoid shapes) and chloroplasts (smaller circular disc like structures) within them. It can be seen that the presence of the herbicide within the cells results in an increase in autofluorescence lifetime and an increase in the heterogeneity of the fluorescence lifetime. In the images shown in Fig. 6d, this heterogeneity is clearly visible across the different FOVs. These images were all acquired at a depth of 100 μm from the leaf surface. Fluorescence decay profiles were fitted to a double exponential decay model, as in [29], using the *FLIMfit* software and the intensity-weighted mean lifetimes (*τ*_*m*_) averaged over the FOV were calculated. This analysis indicated that the untreated FOVs exhibits an average *τ*_*m*_ of 560 ± 30 ps while the treated leaf sample exhibited increased *τ*_*m*_ of 2000 ± 440 ps across different FOVs as shown in Fig. 7, which is consistent with the chlorophyll fluorescence lifetime increasing in the presence of a PS II inhibiting herbicide.

**Fig. 8:**
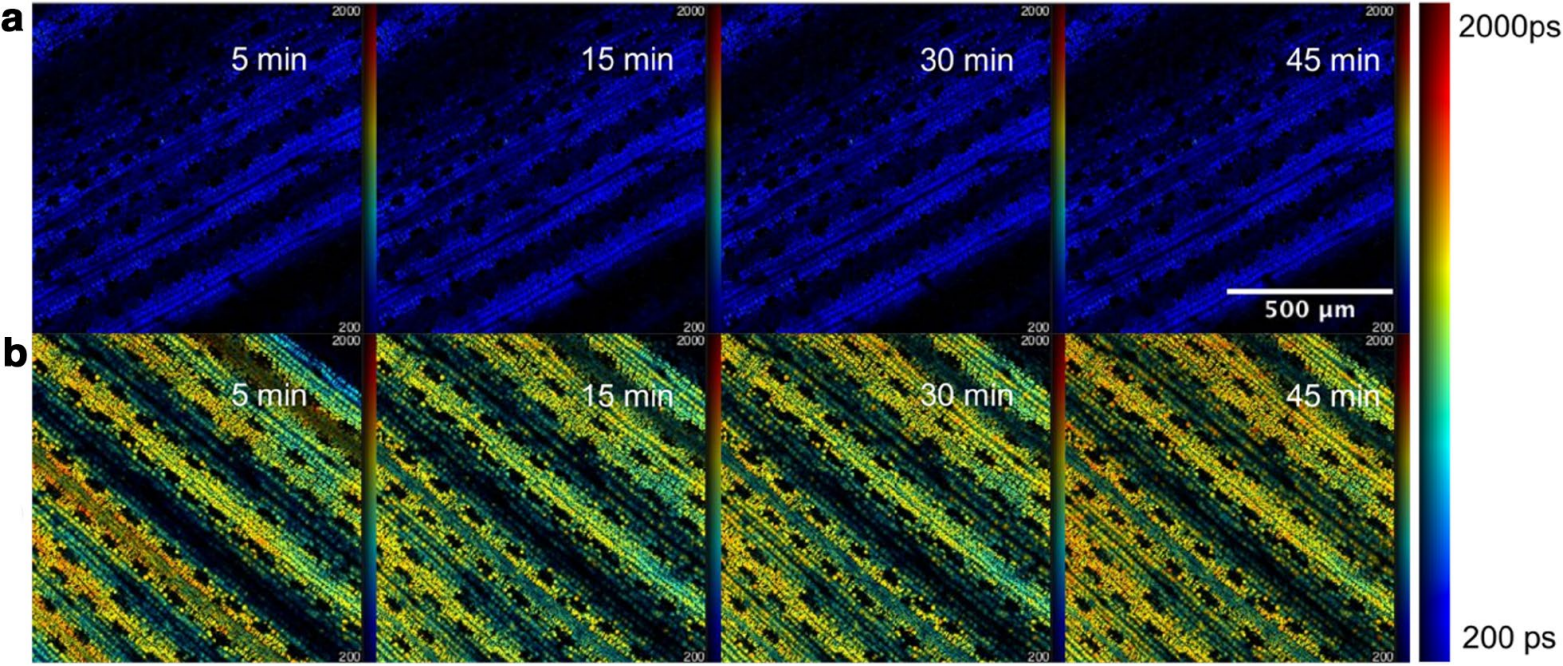
TPE-FLIM images obtained from in vivo time course experiments in *Triticum aestivum* leaves. **a** Shows intensity merged fluorescence lifetime images of an untreated leaf at different time points. **b** Shows intensity merged lifetime images of a treated leaf at different time points showing an increase in lifetime compared to **a**. All the measurements were carried out using a Leica TCS SP5 system and analyzed using FLIMfit software. Imaging was performed using MPE excitation at 900 nm and a ×10 air objective with an NA of 0.30. Each FOV is 1 mm × 1 mm

Following these in situ fixed endpoint measurements, we undertook a time course of two photon excited FLIM (TPE-FLIM) measurements of treated and untreated plants in vivo. Figure 8a shows intensity merged fluorescence lifetime images of (a) an untreated leaf and (b) a treated leaf at different time points. As expected, the treated leaf exhibits an increase in the mean chlorophyll fluorescence lifetime in contrast to that of an untreated leaf.

**Fig. 9:**
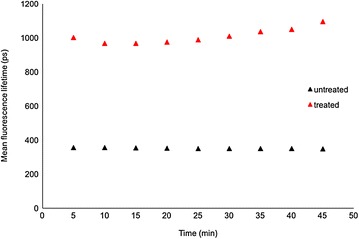
Mean fluorescence lifetime (*τ*_m_) plotted as a function of time for treated and untreated *Triticum aestivum* leaves imaged using multiphoton excitation at 900 nm, see Fig. 8 for corresponding FLIM images. Standard errors are calculated from the pixelwise fit over the field of view shown in Fig. 8

A plot of the weighted mean lifetimes obtained from the analysis is presented in Fig. 9. The untreated leaf control time-course experiment yielded images with a *τ*_*m*_ of 351 (±1 SE) ps averaged over the FOV and the time course of a treated leaf yielded images with an increased mean lifetime of 1021 ps at 5 min to 1110 ps at 45 min post treatment.

### *In vivo* multispectral point-probe autofluorescence lifetime measurements

**Fig. 10:**
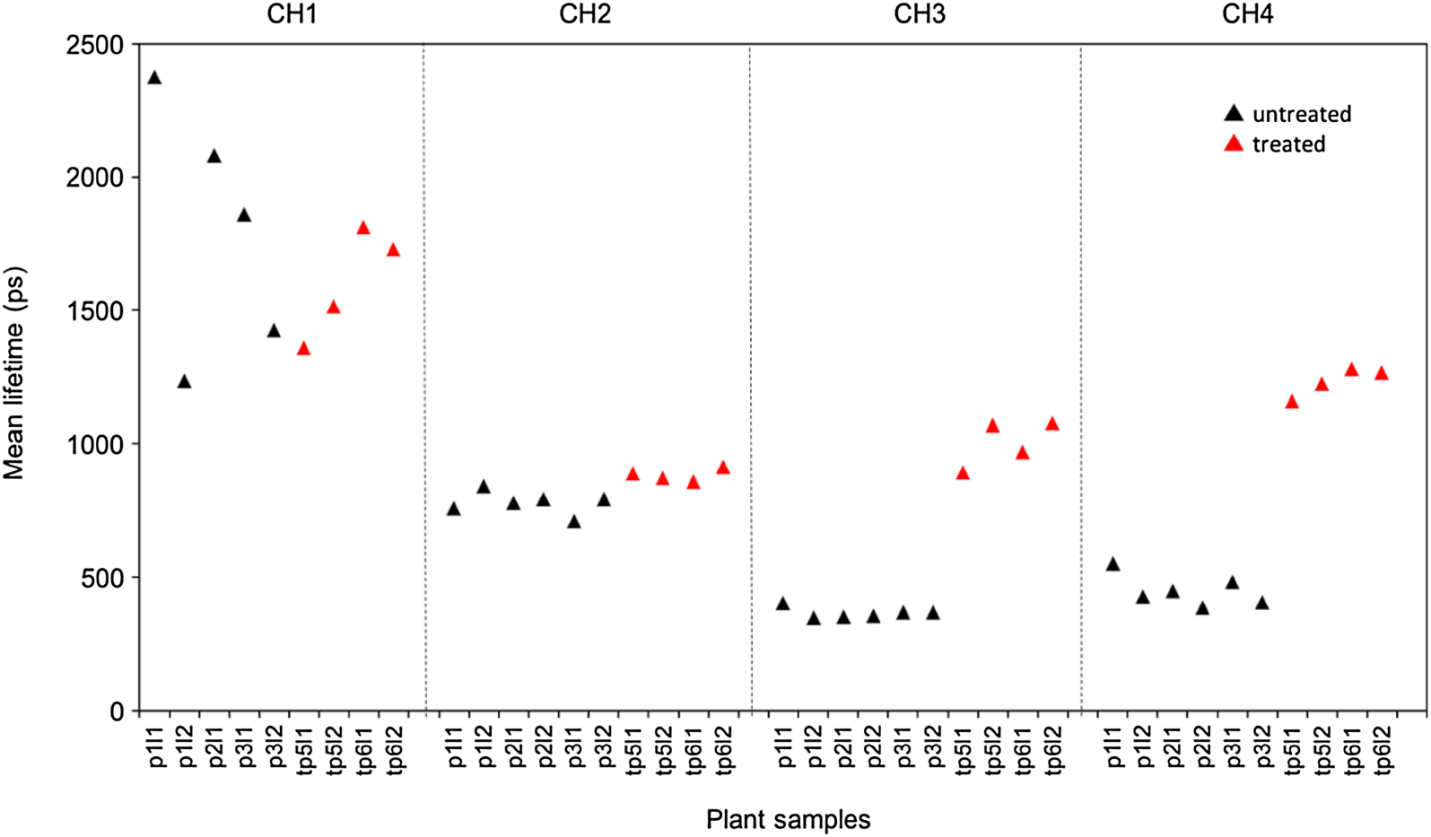
Summary of multispectral lifetime point-probe measurements in *Triticum aestivum*. The different spectral detection channels are represented by CH1, CH2, CH3 and CH4 and are separated by *black dotted vertical lines*. Untreated plant samples are named in the format p(n)l(m), where n is the plant number and m is the leaf number. Treated plants are named tp(n)l(m). The weighted mean fluorescence lifetime (*τ*_m_) calculated for each sample is plotted in the respective spectral channels

The multispectral fibre-optic point-probe was applied to in vivo studies of untreated and treated (with Flagon EC 400) leaves of *Triticum aestivum*, for which the results are shown in Fig. 10. The measured autofluorescence decay profiles were fitted to a double exponential model with the untreated leaves presenting a mean *τ*_*m*_ of 480 (±16 SE) ps in the 440 nm excited chlorophyll channel, CH4 (620–710 nm). For leaves treated with Flagon EC 400, an increase in *τ*_*m*_ up to 1438 ps was observed in this channel.

In CH3, which detects fluorescence (620–710 nm) excited at 372 nm corresponding to the chlorophyll emission wavelengths, an average *τ*_*m*_ of 490(±10 S.E.) ps was obtained for untreated leaves and 1170 (±140 S.E.) ps for treated leaves.

The autofluorescence decay profiles measured in CH2 (430–480 nm, excited at 372 nm) presented a *τ*_*m*_ of ~750 ps for both treated and untreated leaves. This autofluorescence could be attributed to cinnamic acids or lignin [48].

Fluorescence collected from channel CH1 (400–420 nm, excited at 372 nm) is most strongly affected by background fluorescence from the optical fibres and does not follow a definite trend. The variation between measurements is attributed to the low signal level in this channel.

**Fig. 11:**
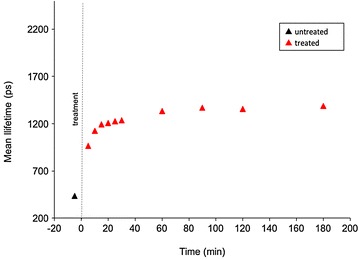
Graph showing the changes in mean lifetime (*τ*_m_) as a function of the time elapsed after treating the plant with Flagon EC 400. *Black dotted line* represents the time of treatment. *Error bars* show standard deviation calculated from 95% confidence interval limits returned by FLIMfit software after fitting the single pixel data

A time course experiment to study the changes in fluorescence lifetime after treating a leaf with Flagon EC 400 was undertaken and also analyzed using a biexponential fit model. The results are shown in Fig. 11. Treatment of the leaf with Flagon was carried out at time = 0 and the time of the “untreated” leaf measurement was 5 min before treatment. In the first 5 min after treatment, *τ*_*m*_ is observed to have increased to 1 ns and then to 1.3 ns after 60 min; thereafter it remained approximately constant up to 180 min. Control experiments where the treatment, i.e. 2 drops of Flagon EC 400, was applied to a black anodized metal sheet resulted in no significant signal detected in the chlorophyll channel, CH4. The resultant fluorescence collected in CH4 from Flagon was found to be insignificant compared to the chlorophyll fluorescence as shown in Fig. 5. We studied the variations in fluorescence lifetimes in untreated lifetime in plants of different age groups and the weighted mean fluorescence lifetimes calculated from CH4 are shown in Additional file 2. It can be seen that there are minor variations from plant to plant and in different age groups, but the general distribution could be seen to be lying in the 350–600 ps range.

### Wide-field FLIM to map herbicide effect in *Triticum aestivum*

**Fig. 12:**
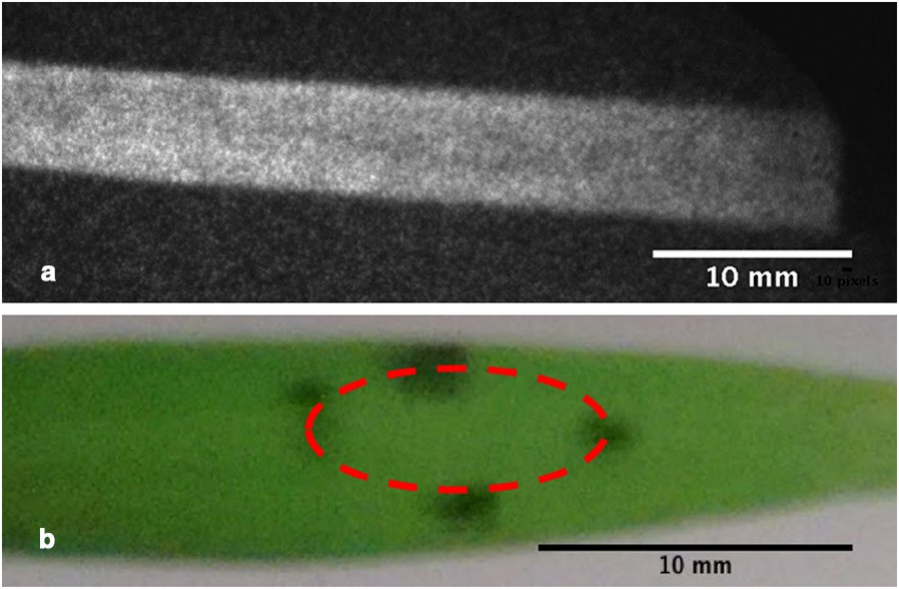
**a** Color photograph of the portion of the leaf sample marked for treatment, the *red dashed ellipse* is a representation of the area to which the treatment spreads once it is applied on to the leaf. **b** Fluorescence intensity image of a leaf recorded by prior to treatment

In vivo wide-field time-gated FLIM of *Triticum aestivum* plant leaves before and following treatment with Flagon EC 400 was undertaken. To orientate this study, Fig. 12a shows a color photograph of the leaf with the treatment area (enclosed by the red-dotted circle) marked with four dots of a black felt pen. Figure 12b shows a fluorescence intensity image of the leaf before it is treated with Flagon EC 400. To confirm that there is no contribution to the fluorescent signal due to fluorescence of the herbicide, we performed a control experiment using the same treatment of Flagon EC 400 applied to a black anodized metal surface and did not detect any significant fluorescence.

**Fig. 13:**
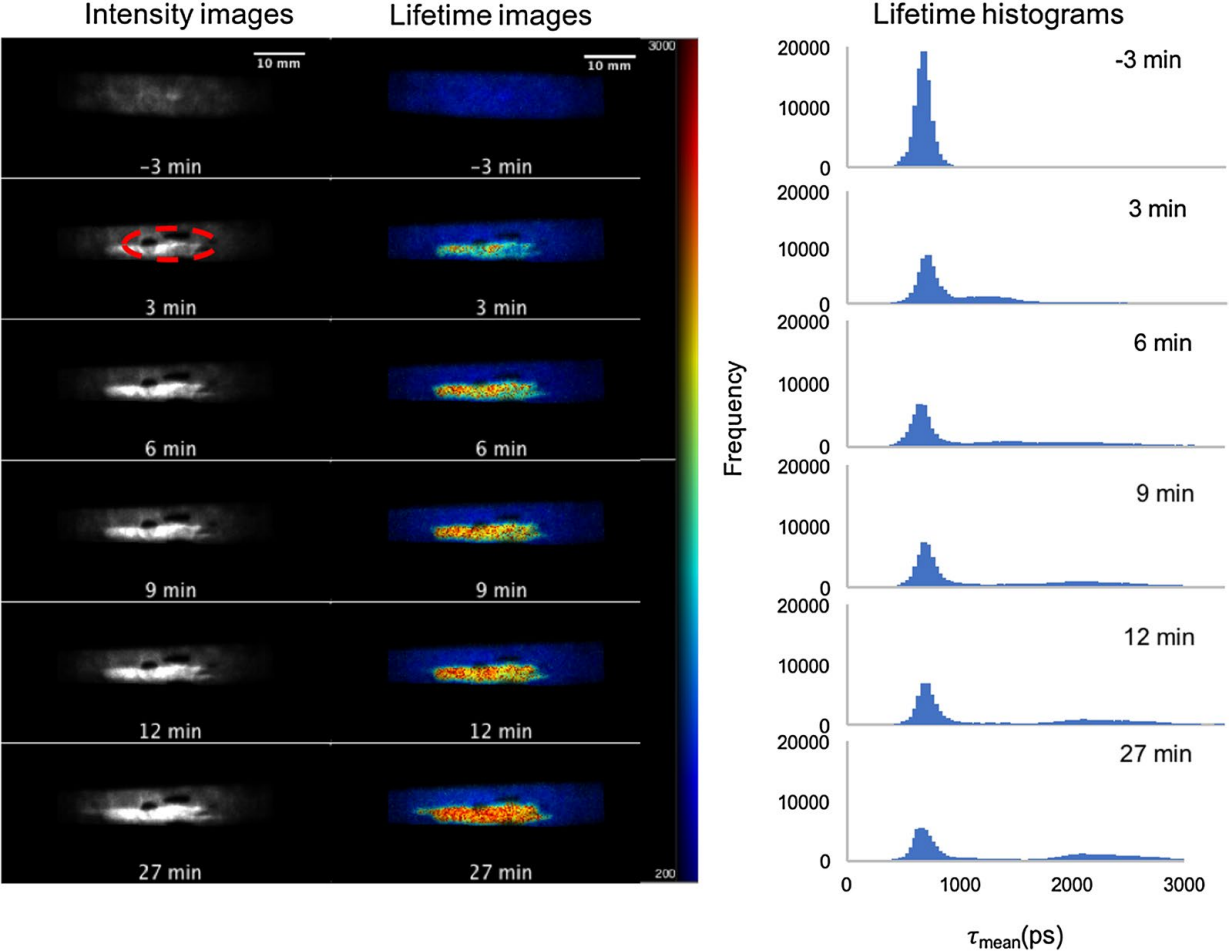
Widefield macroscope images from time course experiments on a leaf. *Each row* shows the fluorescence intensity image, corresponding fluorescence lifetime image and corresponding fluorescence lifetime *histogram* over region of interest for one time point. Images were obtained using the time-gated wide-field macroscope. Images are acquired by exciting the sample at 440 nm (repetition rate 40 MHz) and collected using 9 gates of 1 ns width each. *Red dotted circle* is used to outline the treatment area marked by a black felt pen

**Fig. 14:**
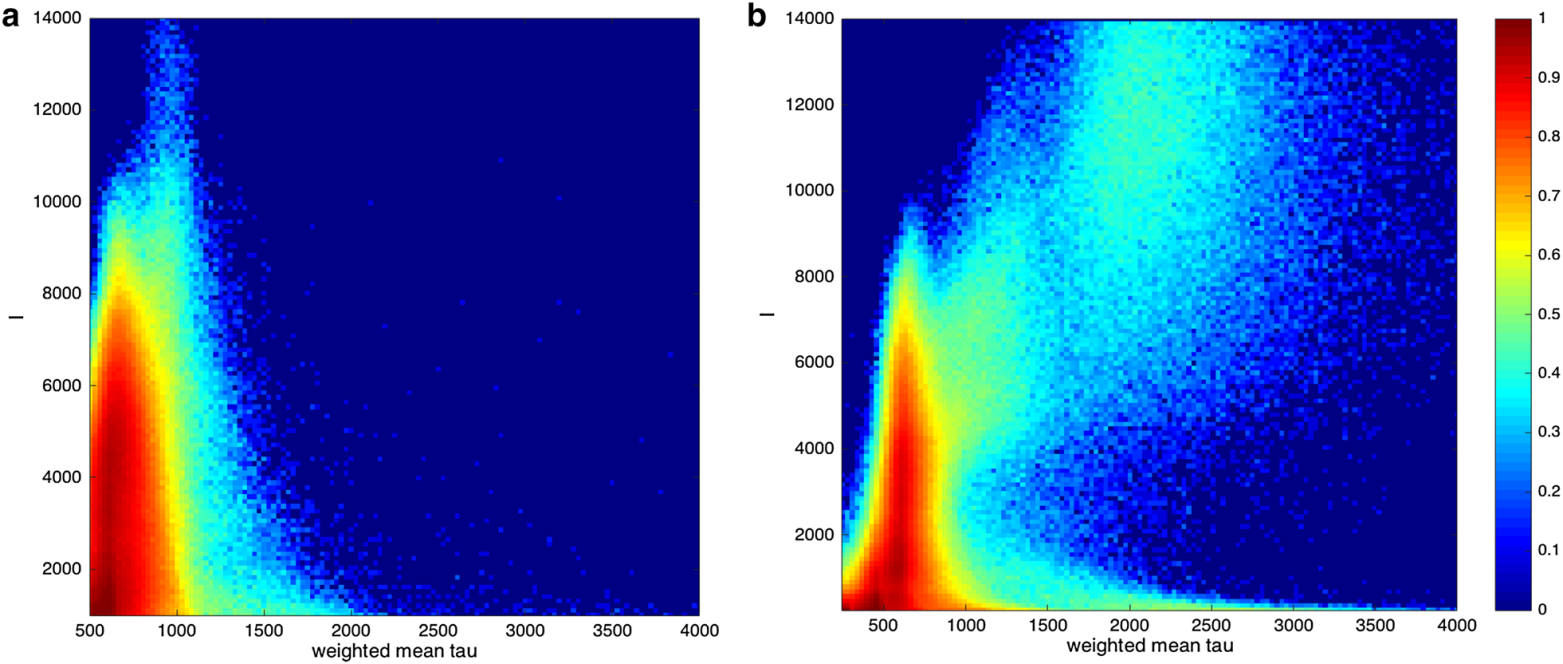
Correlation plot of fluorescence intensity against weighted mean fluorescence lifetime for **a** the untreated region and **b** the treated region from the data acquired from all the time points after application of Flagon EC 400 using the wide field FLIM macroscope (same dataset as shown in Fig. 13)

Wide-field autofluorescence intensity and lifetime images were acquired of a leaf 3 min before treatment and then at 3 min intervals after the treatment up to 27 min. The autofluorescence decay profiles were fitted pixel wise to a double exponential model using the *FLIMfit* software. Time-integrated autofluorescence intensity images and the corresponding intensity-merged autofluorescence lifetime images and lifetime histograms obtained from the experiment at each time point are presented in Fig. 13. The correlation plot shown in Fig. 14 shows 2D histograms of the autofluorescence intensity versus mean lifetime for the treated and untreated regions using the same data set as for Fig. 13. For the pixels with a short mean lifetime (associated with the untreated leaf) the plots show a vertical distribution of points indicating that there is no strong correlation between fluorescence intensity and fluorescence lifetime for this measurement.

**Fig. 15:**
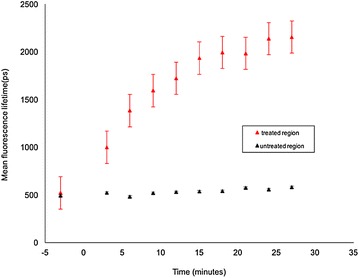
Graph showing the changes in weighted mean lifetime, *τ*_m_ obtained from the wide field macroscope measurements as a function of the elapsed time after treating the plant with Flagon EC 400 for the region of interest marked with *red dotted circles* (treated region) and the region outside it (untreated region) separately. *Black dotted line* represents the time of treatment. Fluorescence lifetime calculations are made separately for the region of interest marked by the *red dotted line* and the region outside it. Standard errors are calculated from pixelwise fit over the region of interest

Figure 15 presents the evolution of *τ*_*m*_ averaged over either the red-dashed region of interest (treated region) indicated in Fig. 12a or the segmented area of the leaf outside the red-dashed region of interest. The leaf presents an initial *τ*_*m*_ of 521 ps before treatment in the red-dashed region of interest. After the treatment with herbicide, the mean autofluorescence lifetime is observed to increase over time, as shown in Figs. 13 and 15. At 3 min after treatment, the mean lifetime was 1000 ps. At 27 min after treatment, *τ*_*m*_ averaged over the red-dashed region of interest was 2150 ps, whereas in the region outside the red dotted circle (untreated region), the fluorescence lifetime remained approximately constant with an average *τ*_*m*_ value of 530 (±10 S.E.) ps.

**Fig. 16:**
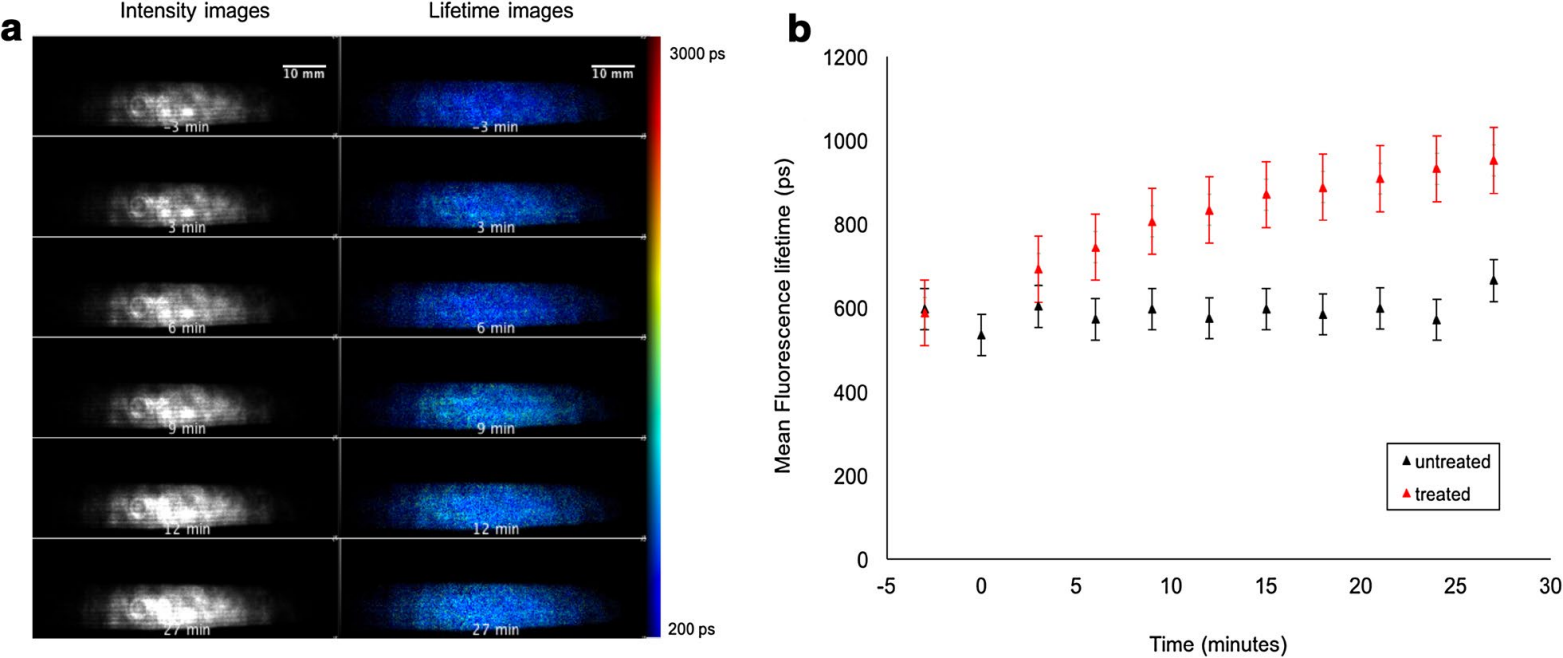
**a** Widefield macroscope images from time course experiments on an untreated leaf. *Each row* shows the fluorescence intensity image and corresponding fluorescence lifetime images at a particular time point. **b** Changes in weighted mean lifetime, *τ*_m_ averaged over the whole leaf obtained from the wide field macroscope measurements as a function of the elapsed time from an untreated life and a separate leaf treated with Flagon EC 400. Fluorescence lifetime values reported are the average over the entire image. Standard errors are calculated from pixelwise fit over the entire field of view

A separate time course experiment was conducted on a different untreated plant and a leaf was imaged under the same conditions and at intervals of 3 min and the results are shown in Fig. 16. The fluorescence lifetimes of the untreated leaf remained fairly constant over a time period of 27 min and yielded an average *τ*_*m*_ of 580 (±10 S.E.) ps, in reasonable agreement with the *τ*_*m*_ values of the untreated regions of the treated leaf.

These lifetime values obtained are in agreement to the multiphoton imaging data. It can be seen from the lifetime frequency histograms that there is a significant decrease in the population fraction of the short lifetime (~580 ps) component and the lifetime distribution shifts towards longer lifetimes (1000–2500 ps) over time after treatment.

Figure 16a shows the evolution over time of maps of the autofluorescence intensity and fluorescence lifetime of an untreated leaf imaged using wide field FLIM. Figure 16b shows the evolution over time of the mean fluorescence lifetime averaged over the whole of the leaf for the treated leaf presented in Fig. 13 and for the untreated leaf presented in Fig. 16a, again demonstrating the observation of a longer autofluorescence lifetime of chlorophyll in herbicide treated leaves.

## Discussion

Although plant autofluorescence is highly complex and is not yet fully understood, this study indicates that it is possible to utilise lifetime measurements of autofluorescence attributed to chlorophyll to read out and map the action of the PS II inhibiting herbicide, Flagon EC 400. Our empirical in vivo measurements across multiphoton microscopy, point-probe lifetime measurements and wide-field FLIM, all support the use of the change in lifetime of the autofluorescence in the chlorophyll emission spectral band to report the presence of the PS II herbicide through its localised impact on chlorophyll fluorescence. In the multiphoton microscopy images shown in Fig. 6d, a heterogeneity of lifetimes is clearly visible across the different FOVs. The *τ*_*m*_ values along the edges of the mesophyll cells are higher (Fig. 6d, especially the second, third and fourth images) compared to the insides of the cells. This could be explained by the active ingredient penetrating the cell membranes and inhibiting the PS II centres, which leads to an increased fluorescence lifetime of chlorophyll molecules in the vicinity of the corresponding herbicide binding sites, i.e. PS II centres. The observed increase in chlorophyll fluorescence lifetime in the presence of a PSII inhibitor may be explained by the blocking of energy transfer to this photosynthetic pathway and the consequently decreased rate of loss of energy through only the radiative and non-radiative decay channels. In principle, it should be possible to relate these observations to previous studies undertaken to model PS II [30] and light harvesting antenna complexes-II (LHC-II) [49, 50] but this would require disentangling contributions from different compartments in the leaf that are integrated in the analysis of our in vivo measurements. Multispectral multiphoton FLIM microscopy may enable this challenge to be addressed by segmenting different compartments in the leaf and will be the subject of future studies.

Our fluorescence measurement spectral windows from ~600 to 710 nm overlap with emission peaks from chlorophyll *a* and chlorophyll *b*. However, since previous studies of energy transfer kinetics of photosynthetic pigments have indicated that chlorophyll *b* molecules transfer most of their excitation energy to chlorophyll *a* in less than 2 ps [51, 52], we believe that our measurements are dominated by chlorophyll *a* fluorescence.

We note that these chlorophyll fluorescence lifetime measurements were undertaken with the leaf in a light-adapted state, for which the different light doses applied using the various methodologies are provided in the Additional file 3: Table S1. This indicates the potential of fluorescence lifetime measurements to provide quantitative readouts of the herbicide presence without subjecting the plant to dark adaptation, which avoids practical difficulties associated with spectral ratiometric measurements that do require dark adaption. We therefore see chlorophyll fluorescence lifetime measurements as a promising methodology for in field measurements.

We note that the distribution of herbicides could, in principle, be mapped by directly imaging the fluorescence of the herbicide but for most herbicides, including Flagon EC 400, this emission is very weak relative to the plant autofluorescence. As indicated in Fig. 3, the fluorescence of Flagon EC 400 is very weak in the chlorophyll detection band and we verified that no significant fluorescence signals were detected from the herbicide for the multispectral point probe measurements and the wide-field FLIM experiments.

## Conclusions

We have demonstrated the in vivo readout of the local action of a PS-II inhibiting herbicide using multiphoton FLIM microscopy, a fibre-optically delivered time-resolved spectrofluorometer for single point lifetime measurements and single photon excited wide-field FLIM applied to leaves of *Triticum aestivum*. We consistently observed an increase in fluorescence lifetime in the chlorophyll autofluorescence detection band following treatment with Flagon EC 400 that can serve as an indirect marker of the herbicide action. The multispectral point probe system already has a form factor suitable to be used as a portable diagnostic tool in greenhouses and in the field. The wide-field FLIM instrument, which enables spatiotemporal mapping of the distribution of this agrochemical over a large FOV comparable to an entire leaf, could also be engineered to be portable for in field studies.

## Additional files

**Additional file 1: Figure S1.** Schematic representation of the optical set-up of multidimensional spectrofluorometer as described in Manning et al. [41].

**Additional file 2: Figure S2.** Distribution of fluorescence lifetimes in untreated *Triticum aestivum* plants of different age groups calculated from multispectral lifetime point-probe measurements in the spectral channel CH4 (excitation at 440 nm, detection wavelengths 620-710 nm). Data points from different age groups are represented by different colours. Plant samples are named in the format p(n)l(m), where n is the plant number and m is the leaf number. The weighted mean fluorescence lifetime (τ_m_) calculated for each sample is plotted here.

**Additional file 3: Table S1.** Table of light doses applied using the various methodologies.

## Abbreviations

PS II: Photosystem II
FLIM: Fluorescence lifetime imaging
SEM: Scanning electron microscopy
CLSM: Confocal laser scanning microscopy
TPEM: Two-photon excitation microscopy
PS I: Photosystem I
DCMU: 3-(3,4-Dichlorophenyl)-1,1-1-dimethylurea
AI: Active ingredient
TCSPC: Time-correlated single photon counting
IRF: Instrument response function
CH1: Channel 1
CH2: Channel 2
CH3: Channel 3
CH4: Channel 4
FOV: Field of view
TPE-FLIM: Two photon excited FLIM
LHC-II: Light harvesting antenna complexes-II
