# Supplementary figures and images for: In vivo label-free mapping of the effect of a photosystem II inhibiting herbicide in plants using chlorophyll fluorescence lifetime
Source: Plant Methods. 2017 Jun 15;13:48. doi: 10.1186/s13007-017-0201-7 (PMC5472976; doi:10.1186/s13007-017-0201-7)

## Slide 1
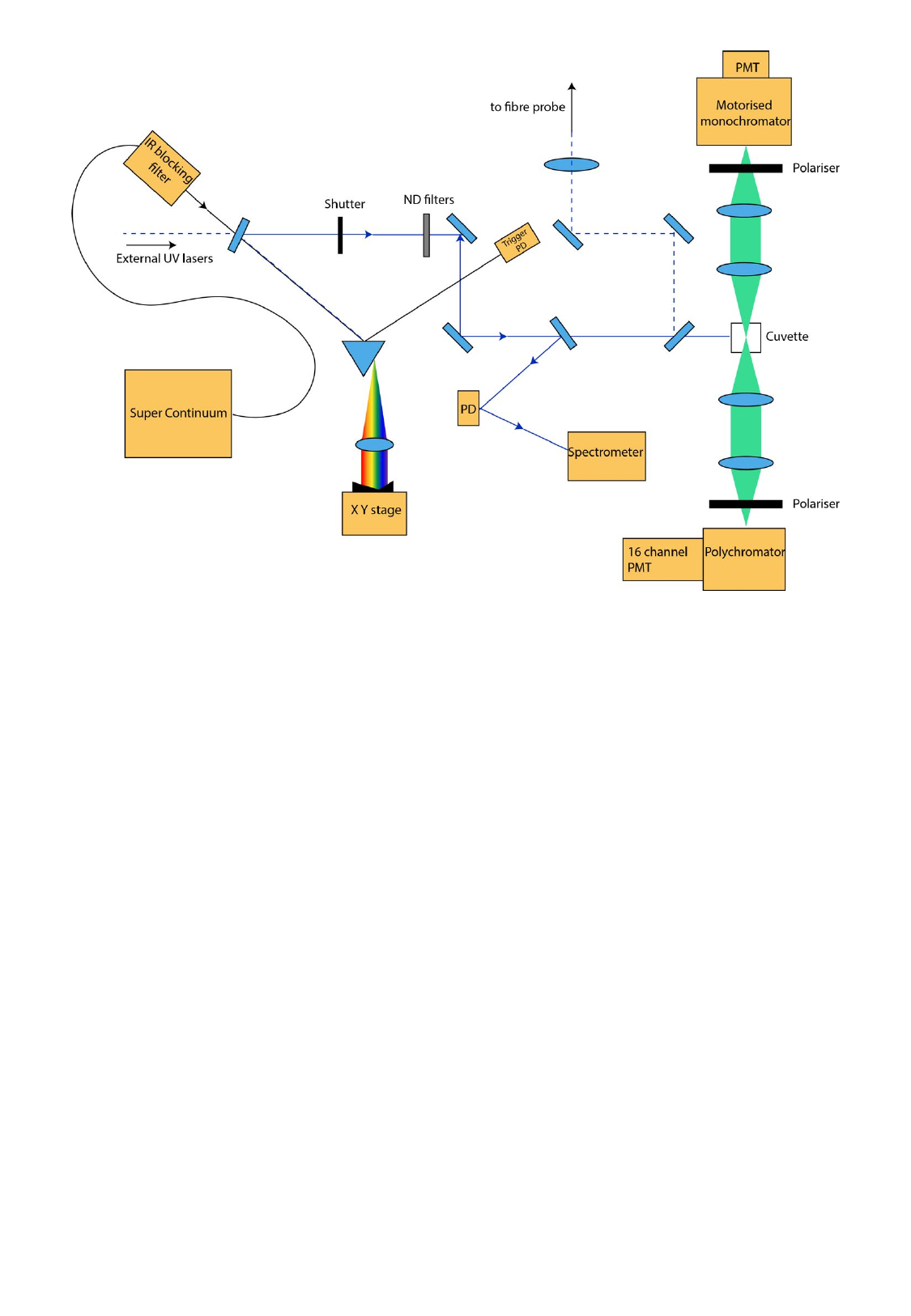

Supplement: Supplementary file 1 — Additional file 1: Figure S1. Schematic representation of the optical set-up of multidimensional spectrofluorometer as described in Manning et al. [41]. [file 13007_2017_201_MOESM1_ESM.pptx]

## Slide 1
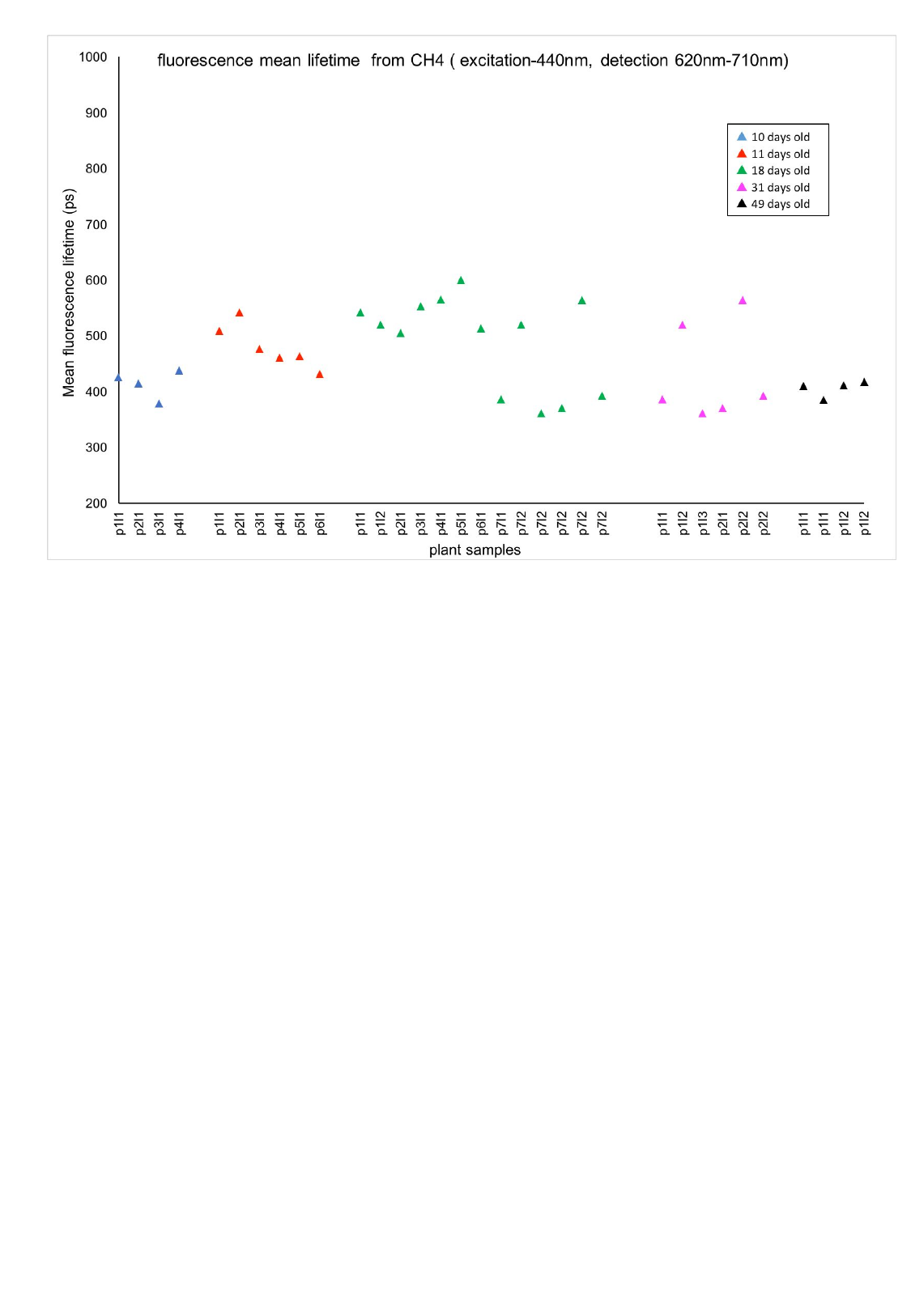

Supplement: Supplementary file 2 — Additional file 2: Figure S2. Distribution of fluorescence lifetimes in untreated Triticum aestivum plants of different age groups calculated from multispectral lifetime point-probe measurements in the spectral channel CH4 (excitation at 440 nm, detection wavelengths 620-710 nm). Data points from different age groups are represented by different colours. Plant samples are named in the format p(n)l(m), where n is the plant number and m is the leaf number. The weighted mean fluorescence lifetime (τm) calculated for each sample is plotted here. [file 13007_2017_201_MOESM2_ESM.pptx]
