# Supplementary material for: In vivo label-free mapping of the effect of a photosystem II inhibiting herbicide in plants using chlorophyll fluorescence lifetime
Source: Plant Methods. 2017 Jun 15;13:48. doi: 10.1186/s13007-017-0201-7 (PMC5472976; doi:10.1186/s13007-017-0201-7)
Supplement: Supplementary file 3 — Additional file 3: Table S1. Table of light doses applied using the various methodologies. [file 13007_2017_201_MOESM3_ESM.docx]

Additional file 3 – Table S1

**Table S1**

| Light source | Average intensity |
| --- | --- |
| Direct Sunlight (250-2500 nm) | 1050 W/m^2^ |
| Photosynthetically active radiation (400-700 nm of sunlight) | 386.4 W/m^2^ |
| Dark adaptation (400-700 nm) | 0.021W/m^2^ |
| Plant grow lights (400-700 nm) | 31.2 W/m^2^ |
| Point probe system (370 nm, 440 nm) | 59 W/m^2^ |
| Multiphoton microscope (900nm) – average intensity over time-lapse image acquisition of 40 s each. | 2.67 kW/m^2^ |
| Multiphoton microscope (900nm) – excitation intensity during single excitation pulse of ~100 fs for a 60s acquisition. | 1.6 MW/m^2^ |
| Wide field macroscope (440nm) | 10 W/m^2^ |
